# Advances in Adipose-Derived Stem Cells Isolation, Characterization, and Application in Regenerative Tissue Engineering

**DOI:** 10.1155/2016/3206807

**Published:** 2016-02-11

**Authors:** Umesh D. Wankhade, Michael Shen, Ravindra Kolhe, Sadanand Fulzele

**Affiliations:** ^1^National Institute of Diabetes, Digestive and Kidney Diseases, NIH, Bethesda, MD 20814, USA; ^2^Duke University, Durham, NC 27710, USA; ^3^Department of Pathology, Georgia Regents University, Augusta, GA 30912, USA; ^4^Department of Orthopedics, Georgia Regents University, Augusta, GA 30912, USA

## Abstract

Obesity is a complex, multifactorial disease that has been extensively researched in recent times. Obesity is characterized by excess deposition of adipose tissue in response to surplus energy. Despite the negative connotations of adipose tissue (AT), it serves as a critical endocrine organ. Adipose tissue is a source of several adipokines and cytokines which have been deemed important for both normal metabolic function and disease formation. The discoveries of metabolically active brown AT in adult humans and adipose tissue derived stem cells (ADSC) have been key findings in the past decade with potential therapeutic implications. ADSCs represent an enticing pool of multipotent adult stem cells because of their noncontroversial nature, relative abundance, ease of isolation, and expandability. A decade and a half since the discovery of ADSCs, the scientific community is still working to uncover their therapeutic potential in a wide range of diseases. In this review, we provide an overview of the recent developments in the field of ADSCs and examine their potential use in transplantation and cell-based therapies for the regeneration of diseased organs and systems. We also hope to provide perspective on how to best utilize this readily available, powerful pool of stem cells in the future.

## 1. Introduction

Regenerative medicine has evolved tremendously with recent advances in stem cell research. The last couple of decades have shown glimpses of the exciting potential of stem cells in tissue regeneration as well as in the restoration of diseased organs and systems. Despite these advances, the availability of stem cells remains a challenge for both scientists and clinicians with an interest in regenerative medicine. In general, a stem cell is characterized by its ability to self-renew and differentiate into multiple lineages. With regard to regenerative medicine, the ideal stem cell population should be present and accessible in abundant numbers, harvestable by a relatively noninvasive procedure, able to differentiate into a variety of cell lineages, easy to transplant to an autologous or allogeneic host, and able to be manufactured in accordance with the currently accepted good manufacturing practice guidelines set by the FDA [1].

The two main types of stem cells are embryonic stem (ES) cells and adult stem cells, also known as somatic stem cells. Other types, such as induced pluripotent stem cells (iPSCs), are produced in the lab by reprogramming adult somatic cells to express ES characteristics. ES cells are obtained by extracting cells from the inner cell mass (ICM) of embryos at the blastocyst stage and subsequently expanding them in culture. ES cells' nature of origin makes them difficult to obtain and raises ethical concerns. In contrast to ES cells, adult stem cells produce only a limited set of specialized cells based on location in a particular tissue. In adults, tissue-specific stem cells are located throughout the body in several tissues. The hematopoietic stem cells found in bone marrow and umbilical cord blood can differentiate into all the different types of blood cells. They are the easiest to isolate and have been used in therapy for decades in bone marrow transplants for diseases such as leukemia, where there is abnormal development of blood cells. Other types of tissue-specific stem cells are usually found deep within tissues and are thus more difficult to obtain and study, especially in humans. The epidermal stem cells continually renew the outer layer of skin, and the epithelial stem cells in the gut similarly continuously replace the gut lining. More recent discoveries include bronchoalveolar stem cells from the lungs of adult humans, which are thought to renew the cellular linings of the lungs [2]. One particularly new and enticing addition to the types of known stem cell sources is the pool of adipose-derived stem cells (ADSCs). ADSCs are found in the perivascular region of white adipose tissue, which include subcutaneous fat deposits [3, 4]. Because of their abundance, ease of isolation, and noncontroversial nature, ADSCs have been considered for applications in regenerative medicine.

This review focuses on isolation techniques for ADSCs, the latest developments in protocols, and therapeutic implications of ADSCs for several diseases. We will also discuss the future directions of ADSCs and promising avenues of research.

## 2. Historical Overview of ADSC

In 2001, a research group at UCLA identified and described a putative population of multipotent stem cells that they termed processed lipoaspirate (PLA) cells due to their derivation from processed lipoaspirate tissue obtained through cosmetic surgery [5]. These stem cells were isolated with the help of enzymatic digestion of the stromal vascular fraction (SVF) of adipose tissue. The SVF can be defined as a minimally processed population of fibroblasts, endothelial cells, smooth muscle cells, pericytes, several types of immune cells, and preadipocytes that have yet to adhere to a tissue culture substrate [6, 7]. Culturing the SVF over time is thought to eliminate many of these cell populations, resulting in an adherent population primarily composed of preadipocytes. However, as Zuk et al. suggested through histology and preliminary PCR analysis, the adherent population derived from the SVF contains a significant number of cells that display characteristics of multipotent stem cells [5]. Specifically, it was proposed that the culturing of the SVF results in a relatively homogenous population of PLA cells that are free of contaminating cell populations and capable of displaying phenotypic characteristics of adipocytes, osteoblasts, and chondrocytes [8]. Moreover, it was demonstrated that clonal cell populations derived from a single PLA cell could also be differentiated into the aforementioned cell types, thus confirming an important prerequisite along the path of identifying a stem cell.

Adipose tissue originates from the mesodermal layer of the embryo and develops partially prenatally, but mostly postnatally [9, 10]. The exact location and developmental history of the adipogenic progenitor cells that constitute stem cells are still unknown, and there is not a standardized nomenclature. In addition, stem cells isolated from different types of adipose tissue depots behave differently [11]. There are two main physiologically distinct types of adipose tissue depots: white and brown adipose tissue, with each performing a distinct biological function. Brown adipose tissue (BAT) is best known for its thermogenic properties, generating heat through the expression of a unique uncoupling protein (Ucp1), which utilizes the mitochondrial proton gradient during oxidative phosphorylation to generate thermal energy rather than energized substrates (ATP). In humans, BAT is present in newborn infants, and the amount of BAT generally decreases as humans age to a point where it is virtually absent in the mature adult. Recently, a few groups have reported the presence of metabolically active BAT in adult humans [12, 13]. White adipose tissue (WAT) is by far the most abundant of two. The principal functions of WAT are to store energy and to provide insulation throughout development. WAT is present in several parts of the body in small depots and can acquire distinct characteristics based on the location of the depot. With the rising epidemic in obesity, characterized by excessive amounts of fat deposition, WAT has received significant attention from researchers worldwide. More recently, WAT has been regarded as playing a multifaceted role as both an energy storage site and an endocrine organ. Secretion of hormones such as adiponectin, leptin, resistin, and other adipokines in addition to several classical anti- and proinflammatory cytokines allows WAT to induce systemic physiological and pathological effects.

Although recent advances have shed light on the functional and physiological differences of different adipose tissue depots, the stem cell content of individual depots is still unexplored. In particular, the stem cell population in WAT may hold greater differentiation potential and multipotency [14]. Furthermore, within the broad category of WAT, it is well established that there are differences between the subcutaneous and omental white adipose depots in human subjects, particularly with respect to preadipocyte and endothelial cell numbers [15]. Because of its volume, visceral adipose tissue may be favored over other tissue types as a source of multipotent cells.

## 3. Isolation Procedure and Characterization

**Figure 1:**
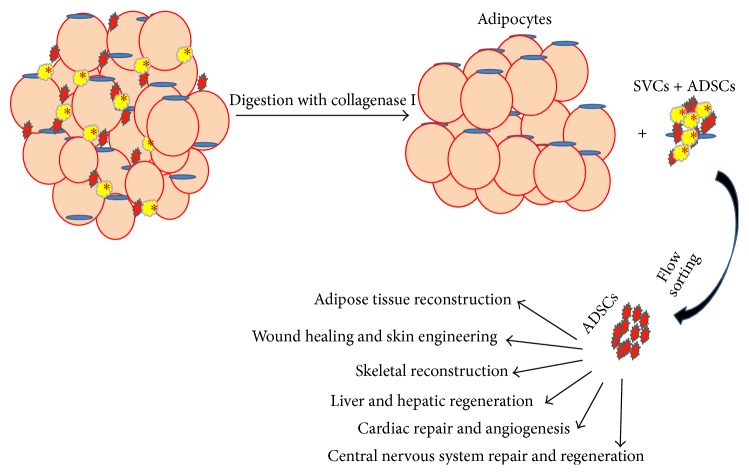
Schematic diagram for ADSCs isolation from adipose tissue and several uses of ADSCs in tissue regenerative medicine.

In 1960s, Rodbell et al. developed a method to isolate cells from adipose tissue [8, 16–18]. They minced rat fat pads, washing them several times to get rid of the contaminating blood and immune cells, and incubated washed tissue fragments with collagenase enzyme to aid in digestion. Following centrifugation, the digested samples would separate into a floating population of mature adipocytes and a pelleted stromal vascular fraction (SVF). The SVF consisted of a heterogeneous cell population, including circulating blood cells, fibroblasts, pericytes, and endothelial cells as well as “preadipocytes” or adipocyte progenitors [8, 18, 19]. After washing with PBS, the SVF population from this stage may be cultured with high FBS (20%) containing DMEM media, with media changes occurring every 12–24 hours to alleviate the effects of collagenase and cell stress (Figure 1).

Adipose tissue is a heterogeneous mixture of several types of cells ranging from immune cells and blood cells to cells of adipogenic origin. To isolate the adipose-derived stem cell (ADSC) population, the cells within the SVF can be categorized using a number of cell type specific markers. The separation of adipocyte precursor populations from nonadipogenic cells using a single cell surface marker is almost impossible [20]. Like most cells, ADSCs are characterized by expression of selective cell surface markers. With the help of multicolor flow cytometry, these putative progenitor cells can be differentiated from nonadipogenic cells such as endothelial and blood cells [21].

An exact phenotypic characterization of ADSCs is still in development, as there is still an unclear distinction between ADSCs, fibroblasts, and several immune cells based on several published protocols. However, adipocyte precursors can be identified by the lack of expression of lineage cell surface markers such as CD45 (blood cell specific) and CD31 (endothelial specific) and characterized by expression of stem cell specific markers including CD29, CD34, and platelet-derived growth factor (Pdgf) receptor-*α*, Sca-1, and CD24 [22, 23]. ADSCs within the SVF putatively express CD31^−^, CD34^+/−^, CD45^−^, CD90^+^, CD105^−^, and CD146^−^; endothelial progenitor populations express CD31^+^, CD34^+^, CD45^+^, CD90^+^, CD105^−^, and CD146^+^; vascular smooth muscle cells (pericytes) express CD31^−^, CD34^+/−^, CD45^−^, CD90^+^, CD105^−^, and CD146^+^; and hematopoietic cells express CD45^+^ in uncultured conditions [24, 25]. ADSCs share many of the same cell surface markers as pericytes and bone marrow-mesenchymal stem cells (MSCs). In addition to the previously mentioned markers, pericyte markers expressed by ADSCs include smooth muscle *β*-actin, pdgfr-*β*, and neuroglial proteoglycan 2 [26]. The MSC markers expressed by ADSCs include CD13, CD29, CD44, CD58, and CD166. Expression of several of these markers varies based on the method of isolation, time of incubation, and stage of growth when in culture. Furthermore, Ong et al. reported differences in surface marker expressions from subcutaneous and visceral adipose tissue depots. ADSCs from subcutaneous adipose expressed higher levels of CD10, whereas visceral adipose-derived ADSCs showed increased expression of CD200 [27]. Because of the overlapping of many cell surface markers, there must be meticulous gate setting while sorting cells via flow cytometry. Further analysis of gene or protein expression with the aforementioned markers can provide additional confirmation.

## 4. Clinical Applications of ADSCs

### 4.1. Adipose Tissue Reconstruction

The use of ADSCs for reconstruction of soft tissue defects has been extensively documented in the literature. Fat injections (FIs), where aspirated fat is reinjected in its entirety without any processing to separate cell populations, have been performed for reconstructive purposes [28]. The most common clinical implication of FIs today is breast soft tissue reconstruction. In the breast, FIs are performed for both cosmetic purposes as in breast augmentation [29, 30] and reconstructive purposes for women with congenital abnormalities or breast cancers in which tissue had to be removed due to metastasis [30–33]. Some studies suggest that supplementation with ADSCs may enhance bulk transplantation procedures such as fat injections. In one triple-blind trial, the survival of fat grafts enriched with autologous adipose-derived stem cells (ADSCs) was compared to the survival of nonenriched fat grafts. Compared with the control grafts, the ADSC-enriched fat grafts had significantly higher residual volumes. These findings support a potential usage of the ADSC stem cell pool in clinical settings. In particular, ADSC graft enrichment could render minimally invasive lipofilling a reliable alternative to major tissue augmentation, such as breast surgery [34]. While adipose tissue within FI contains ADSCs, FIs rely mostly on bulk volume from the transplanted adipose tissue rather than ADSCs to achieve clinical results. As with all transplantation procedures, it is important to consider potential changes in the biochemical environment of the host prior to transplantation. One interesting finding is that the metabolic state of the donor could have an impact on ADSC behavior and expression profile. For instance, ADSCs from obese donors induce significantly greater inflammatory response in the form of higher Th17 promotion and monocyte activation when compared to ADSCs from lean donors. This proinflammatory environment in turn inhibits adipogenesis and the adipocyte insulin response [35]. The demonstration of the ADSCs-Th17-monocyte cell axis suggests a novel proinflammatory process taking place in adipose tissue during obesity [35].

### 4.2. Wound Healing and Skin Engineering

The potential of ADSCs to self-renew and regenerate tissue has great implications in wound healing and skin restoration. Some of the earliest uses of ADSCs in wound healing were in the treatment of chronic fistulae in Crohn's disease with the successful healing of rectovaginal fistula [36–38]. Reportedly, ADSCs were harvested, cultured for up to a month, and then injected directly into the fistula sites with a 75% successful healing rate. The mechanism by which ADSCs aid in wound healing is unclear, but possibilities include direct differentiation of ADSCs within the epithelium, support for angiogenesis in the local tissue, and other paracrine effects from the release of cytokines and growth factors to the region.

Recently, Kuo et al. investigated whether ADSCs can accelerate diabetic wound healing. In a streptozotocin-induced rodent model of diabetes, rats were subcutaneously injected in the diabetic wound margin twice with nondiabetic ADSCs. The ADSC-treated group demonstrated significantly reduced wound healing time compared to the control, and furthermore there was an observed reduction in the proinflammatory characteristics of the ADSC-treated group. There was also an increase in angiogenesis following injection, suggesting involvement of ADSCs in the angiogenesis pathway [39]. In addition to direct transplantation, differentiation of ADSCs to epithelial cells may be another useful application of ADSCs in wound healing. Indeed, there exist varying protocols to differentiate ADSCs to cells with epithelial characteristics using a combination of conditioned media, growth factors, contact with an extracellular matrix, and sometimes specific chemical factors such as retinoic acid. However, the use of ADSCs in skin engineering is still in the experimental stages of development and so far has been predominantly limited to in vitro investigations. Nevertheless, the relative ease of access, high cell yield, and putative anti-inflammatory effects of ADSCs make them attractive targets for skin engineering, in which a large amount of tissue must be supplied for reconstruction [40].

### 4.3. Skeletal Reconstruction

While ADSCs have demonstrated the capability to differentiate into bone and cartilage in the laboratory, the clinical usage of ADSCs for skeletal muscle/bone reconstruction has been limited [5, 41]. The reasons for a lack of translation to the clinical setting may include inefficient differentiation protocols and a limited ability to differentiate into elastic or hyaline cartilage types. One of the earlier instances where ADSCs were used for skeletal reconstruction was attempted bony cartilage healing by Cowan et al. in 2004. ADSCs were seeded into poly lactic-co-glycolic acid (PLGA) scaffolds and were able to repair critically sized cranial defects in mice [42] and in a 7-year-old girl [43]. More recently, a study involved treatment of four patients possessing large cranial defects with a combination of ADSCs and b-tricalcium phosphate granules. Computed tomography (CT) scanning subsequently revealed improved ossification in all of the cases [44]. The efficacy of ADSCs in bone or cartilage based restoration efforts may be improved if ADSCs are used in combination with traditional techniques such as grafts, glues, scaffolds, and supplementation with differentiation factors [45–47].

Because ADSCs are functionally similar to bone marrow derived mesenchymal stem cells, they are capable of directed differentiation to cartilage, muscle, and bone. As mentioned previously, while it seems that ADSCs can successfully generate forms of restored fibrocartilage, the formation of elastic or hyaline cartilage (necessary for joint reconstruction) has not been successfully demonstrated [48]. For other forms of cartilage, however, a recent study suggests that larger doses of growth factors and shear stressing can be beneficial to chondrogenesis during ADSC transplantation [49, 50]. In addition to cartilage, ADSCs can undergo differentiation to form muscle cells. There are a number of protocols for the myogenic differentiation of ADSCs, most of which focus on upregulating expression of the transcription factor MyoD. One recent finding may point to the importance of cell markers in determining myogenic potential among ADSCs. Specifically, Desiderio et al. reported that NG2^+^ADSCs loaded on XHA scaffolds can be fabricated into human skeletal muscle tissue in vivo without a prior myogenic differentiation step in vitro. While NG2^+^ ADSCs were able to differentiate into muscles, NG2^−^ ADSCs merely formed human adipose tissue [51]. For osteogenic differentiation, culture and growth conditions seem to be particularly important. McCullen et al. showed that elevating extracellular calcium from a concentration of 1.8 to 8 mM accelerates human ADSC osteogenic differentiation and cell-mediated calcium accretion, even in the absence of any other soluble osteogenic factors in the culture medium [52]. Recently the same group reported that elevated calcium induced osteogenesis and inhibited chondrogenesis in human ADSCs. Based on these findings, stacked polylactic acid nanofibrous scaffolds containing either 0% or 20% tricalcium phosphate nanoparticles were electrospun and tested for site-specific chondrogenesis and osteogenesis [53]. Approaches such as changing the ionic concentrations and culture conditions hold great promise for osteochondral tissue engineering using an ADSC cell source in combination with a scaffold. In general, optimizing the physical and chemical microenvironment of ADSCs during transplantation may hold the key to making clinical progress in connective tissue regeneration.

### 4.4. Liver and Hepatic Regeneration

Evidence suggests that ADSCs can differentiate into cells with hepatocyte-like characteristics under appropriate culture conditions and that transplantation of ADSCs can improve hepatic function [54]. While the precise mechanisms of these functional changes are still not clear, these results suggest that ADSCs may be useful for the regeneration of the liver. Injection of human ADSCs into the tail vein of mice has been shown to lead to multiorgan engraftment of the cells [55]. Two months after injection, the mice demonstrated the presence of human-derived cells in the bronchus of the lungs, the gastrointestinal tract, and the liver. Interestingly, the liver held a significant proportion (6.9%) of ADSC occupancy whereas no human cells were found in other tissues, such as skeletal and cardiac muscle, kidneys, or skin. These findings suggest a unique interaction of ADSCs with hepatocyte populations and could suggest a potential role of ADSCs in hepatic regeneration. Recently, Tang et al. showed that basic fibroblast growth factor treatment enhances the therapeutic effect of ADSCs, and secretion of hepatocyte growth factor from ADSCs plays a critical role in amelioration of liver injury and regression of fibrosis [56].

### 4.5. Cardiac Repair and Angiogenesis

Cardiac muscle is considered to be a tissue with very limited capacity for self-renewal and repair. In particular, there is an inability to naturally and adequately replace damaged myocardium in cardiovascular complications such as chronic cardiac disease and myocardial infarction (MI) [57]. Given the prevalence of heart diseases and their associated complications, alternative cell-based therapies enlisting the aid of ADSCs and other forms of MSCs are being tested for regenerative potential. In 2004, a research group led by Planat-Bénard identified a very small subpopulation (0.02–0.07%) of cardiomyocyte-like cells in murine SVF which exhibited contractile behavior at day 24 following primary culture [58]. These cells were positive for several cardiac-specific genes, including GATA-4 and Nkx2.5. Later, in 2007, Song and colleagues from the United States showed a similarly small (0.005–0.07%) subpopulation of spontaneously beating cells in human SVF [59]. Coadministration of control-released *β*-FGF along with ADSCs led to angiogenesis and vessel maturation in a murine ischemic hind limb model. This was partly assisted by secretion of hepatocyte growth factor, vascular endothelial growth factor, and transforming growth factor *β*1 [60].

De Francesco et al. reported different cell populations expressing mesenchymal stem cell markers, such as CD34, CD90, CD29, CD44, CD105, and CD117, and endothelial-progenitor-cell markers, including CD34, CD90, CD44, and CD54, present in SVF. Notably, the CD34^+^/CD90^+^ expressing cells formed sphere clusters and demonstrated high proliferative capacity when placed in nonadherent growth conditions. When cultured in an adipogenic medium, CD34^+^/CD90^+^ cells quickly differentiated into adipocytes and also into endothelial cells (CD31^+^/VEGF^+^/Flk-1^+^). When placed in methylcellulose, these cells were further capable of forming capillary-like structures producing a high level of VEGF [61, 62].

The clinical usage of ADSCs in the treatment of chronic cardiac disease or acute MI in humans is still quite distant. Elucidating cellular markers associated with cells possessing particularly high affinity to differentiate into cardiomyocytes may be a promising avenue of research. ADSCs are known for their strong proliferative capacity and express high levels of hyperpolarization-activated cyclic nucleotide-gated (HCN) genes, which code for ion-gated HCN channels. These channels are essential for generating rhythmic activity within clusters of cardiac cells. Friis and colleagues in Denmark have published a safety and feasibility study looking at intramyocardial injection of cultured BMSCs in patients with stable CAD [63]. They treated 31 patients and concluded that the treatment was safe and resulted in significant improvements in left ventricular function and exercise tolerance. Overall, there is a growing body of evidence in laboratory and animal models that ADSCs could improve cardiac function via cell differentiation and paracrine pathways [57, 64]; we anticipate exploration with human trials in the coming years.

### 4.6. Central Nervous System Repair and Regeneration

There is a paucity of literature showing the potential of ADSCs to differentiate into neuronal lineage cells; however, there have been a few documented instances where ADSCs can acquire neuron-like characteristics under appropriate growth conditions. If efficient neurogenesis is possible, ADSCs could potentially provide a huge boon to the current clinically viable options to regenerate nerves and neural tissue. Endogenous neural stem cells (in the central nervous system) and Schwann cells (in the peripheral nervous system) are difficult to harvest and accordingly have limited applicability [65]. Morphological changes towards a neuron-like appearance have been reported for ADSCs when treated with individual or multiple growth factors such as BNDF, EGF, and *β*FGF. A variety of specific chemical stimuli have also demonstrated abilities to induce such morphology. For instance, ADSCs can be transdifferentiated into neuron-like cells using selegiline, as evidenced by an increase in the number of nestin and NF-68 immunoreactive cells following treatment [66]; however, chemically induced morphological changes may not represent actual cellular differentiation, but rather cell shrinkage. Thus, it is important to characterize the extent of neuronal differentiation of ADSCs using known neuronal markers such as vimentin, nestin, GFAP, and MAP-2, in addition to morphology. Other stimuli or conditioning media may be used to further enhance the acquisition of neuronal characteristics by ADSCs. For instance, one group recently showed that human ADSCs could be differentiated in rat brain-conditioned media towards neuron-like cells with a similar efficacy as bone marrow stem cells (hBMSCs); however, because of their higher proliferation rate and greater availability, the authors suggest that ADSCs may be a more viable option for future cell-based neuroregeneration efforts [67].

## 5. Miscellaneous Use

It has been demonstrated that an intravitreal injection of ADSCs can stabilize the retinal microvasculature, enabling repair and regeneration of damaged capillary beds in vivo. Interestingly, one report suggests that diabetic ADSCs are functionally impaired compared to healthy ADSCs and supports the utility of an allogeneic injection of ADSCs versus autologous or conditioned media for approaching the treatment of diabetic retinopathy [68]. Urinary bladder regeneration is another area where ADSCs could play an important role. ADSCs have been successfully used on a PLGA scaffold to reconstruct half of a bladder wall in a nude mouse model [69] in addition to two human patients [70]. This may be very encouraging news for diseases such as urinary stress incontinence and could lead to new cell-based therapy incorporating ADSCs. However, in contrast to these findings, another group showed no difference between fat and saline injections in the treatment of female urinary incontinence at 3 months' follow-up and suggested that the fat may not be able to survive at the injection site [70]. Thus, more work is needed to determine the applicability of ADSCs in this area.

Human and rodent ADSCs can be stimulated to differentiate towards the osteogenic lineage [71, 72]. In vivo, ADSCs differentiate into bone when implanted ectopically into rodents. Rat-isolated ADSCs seeded in polyglycolic acid will begin bone formation when implanted subcutaneously [73, 74]. Similarly, human ADSCs in HA-TCP scaffolds differentiate to osteocytes in immunodeficient mice [75, 76]. Several preliminary reports have also shown the promising results of ADSC treatment in Crohn's disease [77]; however, many of these putative therapeutic applications of ADSCs need to be validated with further research.

## 6. Concerns and Safety Issues with Adipose-Derived Stem Cells

The use of stem cells to treat different maladies has gained popularity over the past decade. ADSCs have demonstrated tremendous potential because of their availability, plasticity, and versatility with regard to regeneration of different tissues. However, despite some clinical trials in humans and existing data from animal experiments, the various risks and safety concerns associated with ADSC transplant have not been fully elucidated. Since adipose tissue serves important auxiliary endocrine functions, transplantation of ADSCs may exert unintended paracrine and endocrine effects on peripheral tissues. For instance, the secretion of metabolically active hormones such as leptin, cytokines such as TNF-*α* and IL-6, and several growth factors could disrupt peripheral biochemical pathways [78]. Recently Eljaafari et al. reported that obese- but not lean-derived ADSCs induce a proinflammatory response mediated through Th17 promotion and monocyte activation, which leads to inhibition of adipogenesis and adipocyte insulin response [35].

One important consideration with every stem cell pool is the potential for tumorigenicity upon transplantation. The aforementioned production of IL-6 by ADSCs is concerning, since IL-6 is known to be a crucial cytokine in the induction and progression of tumors. Other proinflammatory and chemokine factors such as IL-8, MCP-1, RANTES, TGF-*β*, and VEGF belong to the expression profile of ADSCs while also being markers associated with tumor growth and metastasis; however, the relationship between ADSCs and cancer cells is not fully understood, as evidenced by mixed reports in the literature. In one animal model, ADSCs were shown to aid in chronic inflammation and assisted the transformation of gastritis to gastric cancer [79]. By contrast, another study showed that ADSC implantation actually inhibited breast cancer development in mice [80]. Additionally, ADSCs ostensibly secrete many factors related to the epithelial-mesenchymal transition (EMT), an important step in tumor metastasis; however, a discord still exists between basic science and clinical findings [81]. The applicability of these animal studies to humans undergoing clinical treatment remains doubtful. It is entirely possible that the interaction between ADSCs and tumor cells may be entirely characteristically different in humans, especially considering the differences in adipose tissue depot composition and location between humans and rodents. Thus, further basic studies on the interaction between hADSCs and various cancers will need to be performed to elucidate any potential hazards such as de novo tumorigenesis or the reinforcement of existing tumors. A series of well-designed and controlled clinical trials will be necessary.

## 7. Future Directions of Adipose-Derived Stem Cell Therapies

The potential of adipose tissue to be a prolific source of multipotent adult stem cells has garnered a great deal of attention in the field of regenerative medicine. With the increasing number of overweight and obese individuals, isolation of ADSCs from lipoaspirate samples may prove to be a clinically feasible option. Given the relative abundance of ADSCs, their ease of harvest and culture, and their high yield relative to other stem cell pools such as bone marrow, it is likely that the research and clinical usage of ADSCs will continue to grow. While some initial reports show positive clinical outcomes, well-designed and controlled studies as well as long-term posttreatment follow-ups will be paramount to ensure the safety and efficacy of these procedures for patients.

Stem cells have proven to be a useful tool in cell-based therapies for a wide collection of diseases. For ADSCs, the development of detailed and efficient differentiation protocols for various cell types, optimization of in vivo delivery methods, and mitigation of immune response in allogeneic transplantations are some of the challenges that need to be overcome. Many of these challenges have been considered and investigated of late, but additional work is necessary in order to bridge the gap between findings in basic science and the clinical treatment of diseases with stem cell-based regenerative medicine.
